# Assembly and Maturation of a *T =* 4 Quasi-Equivalent Virus Is Guided by Electrostatic and Mechanical Forces

**DOI:** 10.3390/v6083348

**Published:** 2014-08-22

**Authors:** Bradley M. Kearney, John E. Johnson

**Affiliations:** Department of Integrative Structural and Computational Biology, The Scripps Research Institute, La Jolla, CA 92037, USA; E-Mail: bmkearne@scripps.edu

**Keywords:** virus maturation, quasi-equivalence, *Nudaurelia capensis* omega virus, autoproteolysis, tetravirus, RNA insect virus, non-enveloped viruses

## Abstract

*Nudaurelia capensis* ω virus (NωV) is a eukaryotic RNA virus that is well suited for the study of virus maturation. The virus initially assembles at pH 7.6 into a marginally stable 480-Å procapsid formed by 240 copies of a single type of protein subunit. During maturation, which occurs during apoptosis at pH 5.0, electrostatic forces guide subunit trajectories into a robust 410-Å virion that is buttressed by subunit associated molecular switches. We discuss the competing factors in the virus capsid of requiring near-reversible interactions during initial assembly to avoid kinetic traps, while requiring robust stability to survive in the extra-cellular environment. In addition, viruses have a variety of mechanisms to deliver the genome, which must remain off while still inside the infected cell, yet turn on under the proper conditions of infection. We conclude that maturation is the process that provides a solution to these conflicting requirements through a program that is encoded in the procapsid and that leads to stability and infectivity.

## 1. Introduction

**Figure 1:**
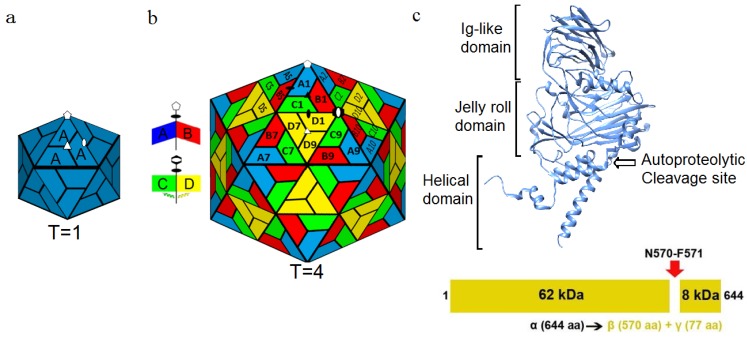
Icosahedral arrangement of capsid proteins. (**a**) The *T =* 1 surface lattice where 60 copies of a single gene product are used to form a complete capsid. White symbols identify icosahedral 5-fold (pentagon), 3-fold (triangle), and 2-fold (ellipse) symmetry axes; (**b**) The *T =* 4 surface lattice seen in NωV where an asymmetric unit containing 4 copies of a single gene product forms the icosahedron (240 total protein subunits). Local environments allow for quasi-symmetry in addition to the standard icosahedral symmetry elements. Dimer interfaces at the quasi-2-fold axes occur with either bent (A–B) or flat (C–D) conformations. White symbols identify icosahedral symmetry axes, and black symbols identify quasi-2-fold (ellipses) and quasi-3-fold (triangle) axes. The hexagon with the white ellipse embedded identifies a quasi-6-fold symmetry axis (icosahedral 2-fold axis); (**c**) Schematic of the α protein that makes up the capsid. The capsid protein is comprised of the N-terminal and C-terminal helical domain which interacts with the RNA, the Jelly roll domain and the Ig-like domain on the surface of the capsid. An autoproteolytic cleavage site (N570-F571) in the helical domain is activated during maturation, yielding the β protein and γ peptide. Reproduced with permission from Veesler, D. and Johnson, J.E. [2].

Viruses evolved to be exquisitely tuned machines that optimize structure and function. The genetic payload of the simplest viruses is enclosed in a genetically economical capsid, formed by multiple copies of a single type of gene product encoded by the viral genome. The icosahedron, formed by 60 identical asymmetric units, encloses the maximum volume for a given sized asymmetric unit and readily explains why many viruses, including a large number of important human pathogens [1], display the symmetry of an icosahedron. Icosahedral capsids formed by 60 subunits place all the proteins in identical environments (Figure 1a). A virus can package larger genomes with larger protein subunits or with multiple proteins (either the same or different gene products) in the icosahedral asymmetric unit (Figure 1b).

### 1.1. Quasiequivalence

Simple logic, based on the subunit mass and the particle size, demonstrated that the first plant viruses studied by electron microscopy and X-ray diffraction contained more than 60 subunits, yet displayed icosahedral symmetry. The geometric explanation for these particles was derived by Caspar and Klug [3] and is based on the principles employed by Buckminster Fuller to build geodesic domes [4]. They showed that these, so called, quasi-equivalent capsids contain 60 T subunits where *T =* h^2^ + hk + k^2^ and h and k are positive integers. Viruses that exhibit quasi-equivalence possess true icosahedral symmetry, but have additional symmetry elements that only hold in local environments [5]. Local symmetry is generated by addition of hexamers (following specific selection rules) into an icosahedral surface lattice. The rationale for hexamers formed by the same subunits that form pentamers relates to the small difference in rotation between the subunits (60 degrees *versus* 72 degrees), thus hexamers and pentamers are quasi-equivalent to each other and, with that assumption, quasi 2-fold and 3-fold axes are also generated (Figure 1b). In principle nearly the same interface can be maintained if the hexamers form a flat surface and pentamers are canted upward. This also suggests differentiation of planar and curved regions associated with hexamers and pentamers respectively. Caspar and Klug originally envisioned quasi-equivalence being accommodated by the intrinsic flexibility of the protein surfaces that would allow the adjustment of subunit interfaces to accommodate 5 and 6-fold symmetry. However, most quasi-equivalent capsids studied have modular subunits with rigid folds in one portion and dynamic N and/or C terminal portions that exhibit conformational polymorphism that switches subunit interface interactions and hence the quaternary structure. The local environments, coupled with conformational polymorphism, result in polypeptide regions formed by the same amino acid sequence performing different roles.

This description holds for mature capsids but provides no mechanistic explanation for how the observed structural polymorphism is achieved. The next section provides a conceptual model for achieving this remarkable result.

### 1.2. Assembly of Provirions

**Figure 2:**
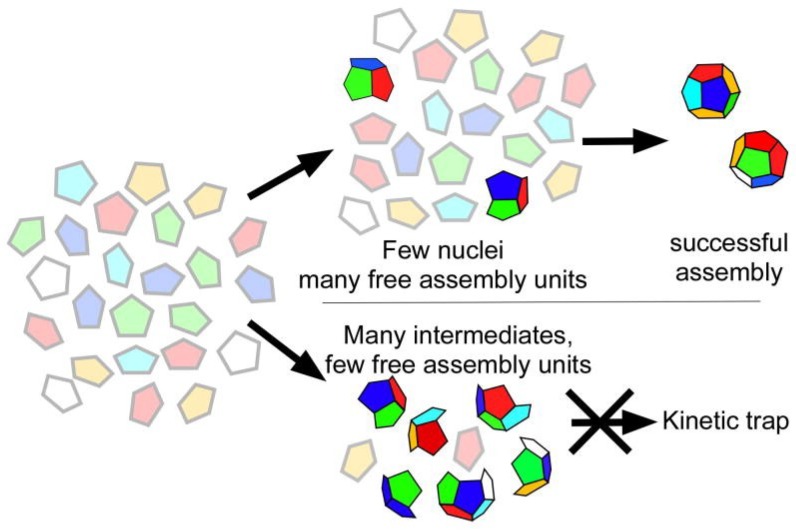
Successful assembly and kinetic traps. Under conditions that force quasi-symmetry too early and promote rigid associations, a majority of the assembly products will be malformed. By allowing for gentle assembly where subunits are not forced to differentiate during the procapsid phase, successful assembly is promoted**.** Reproduced with permission from Zlotnick, A. and Mukhopadhyay, S. [6].

Quasi-equivalence requires that identical gene products reside in different geometric environments in an icosahedrally symmetric shell, an outcome that is not obvious. A simplistic model for a generic, quasi-equivalent virus envisions subunits in equilibrium between pentamer and hexamer states in solution, with possibly a basic portion of the subunit interacting with the nucleic acid that contributes to genome packaging and protein nucleation. Hexamer and pentamer capsomers interact with one another through Brownian motion and are stabilized in their oligomeric form by the contacts. Following the formation of an initial hexamer–pentamer pair, additional hexamers and pentamers are added at proper positions by subtly encoded surface preferences. Throughout, the interactions must be just on the assembly side of a dynamic equilibrium (~2–4 kT) with annealing and self-correcting a critical component of the process [6]. Procapsid assembly intermediates are intrinsically dynamic and relatively unstable, although the successful addition of oligomers will increase the overall stability to a point where the procapsid can exist in the cellular environment in which it assembles. Stronger interactions during the initial assembly process would not be productive since the amount of viable capsid produced would be outweighed by dead-end products that fell into kinetic traps (Figure 2) [6].

The final assembly product described is generically referred to as a provirion. Such particles have been extensively studied for dsDNA bacteriophage and enveloped viruses such as Dengue virus and HIV. Generally they are not infectious because they require additional steps to gain infectivity and stability. These steps are referred to as maturation.

### 1.3. Maturation

Maturation is a remarkable process in which the particle transitions from usually a spherical, moderately stable procapsid assembly product to a robust infectious virion. Double stranded DNA packaging triggers bacteriophage capsid maturation, while in HIV, activation of a virally encoded protease is critical. Protease inhibitors, a major therapy for HIV infected individuals, prevent this maturation and infection. An environmental cue invariably triggers maturation and it occurs late in the infection, just prior to or immediately after the provirion leaves the cell. The study of virus maturation allows the investigation of a chemically encoded program that resides in the procapsid and that is executed following the environmental cue. Subunits may undergo large-scale reorganization, with molecular switches differentiating quasi-equivalent subunits into distinct environments that were closely similar in the procapsid. Often there is an autocatalytic cleavage that activates a viral entry mechanism and the infectious virion is tuned to be sufficiently stable to move cell to cell or to survive in the ex vivo environment and yet, following interaction with an appropriate receptor, destabilizes again for the release of its genome into the appropriate location in the cell.

Tetraviruses, the subject of this review, are the most accessible non-enveloped animal virus identified to date for the biochemical and biophysical study of virus maturation and this motivates their use to study fundamental aspects of virus maturation.

## 2. Tetraviruses

Tetraviruses initially gained the interest of the scientific community 50 years ago due to their ability to cause epizootics in pest insect species [7]. These viruses exclusively infect Lepidopterans and are species specific, rarely infecting more than a few closely related species [8]. Tetraviruses are non-enveloped and have single-stranded positive-sense RNA genomes. They are subdivided into two genera based on the genome structure. Betatetraviruses, such as *Nudaurelia* *capensis* β virus (NβV) [9], *Thosea asigna* virus (TaV) [10], and Providence virus (PrV) [11], have monopartite genomes of around 6.5 kb that encode the RNA-dependent RNA polymerase (RdRP) and, through a subgenomic RNA, the capsid protein [9]. Omegatetraviruses, such as *Nudaurelia* *capensis* ω virus (NωV) [12], and *Helicoverpa armigera* stunt virus (HaSV) [13], have bipartite genomes with the RdRP encoded on RNA1 (~5.3 kb) and the capsid protein encoded on RNA2 (~2.5 kb).

**Figure 3:**
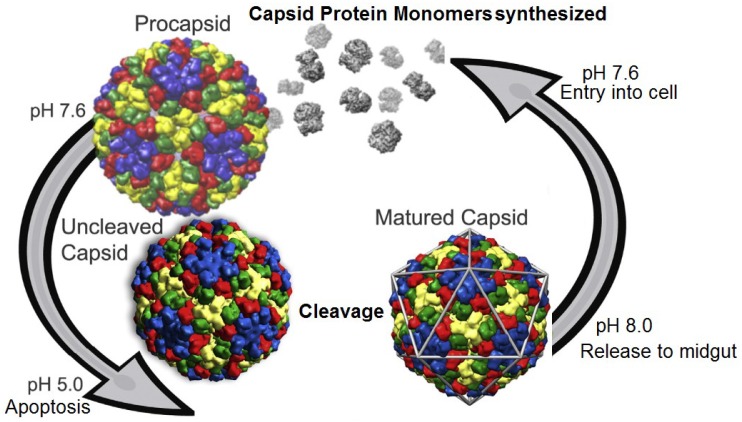
Life cycle of tetraviruses. Infected cells produce procapsids at relatively neutral pH. Production of virus particles eventually triggers apoptosis which induces a drop in pH and virus maturation. Release to the alkaline midgut allows the virus to further infect cells and start the cycle anew. Reproduced with permission from Domitrovic *et al*. [15].

**Figure 4:**
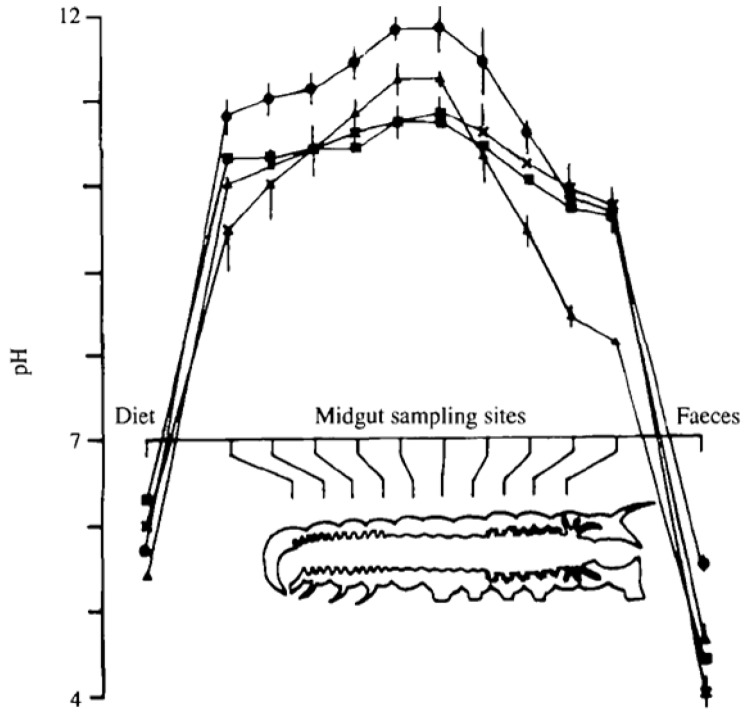
pH profile of the midgut of Lepidopteran larvae. The midgut is highly alkaline along the entire path of the midgut and it is in this environment that the virus infects new cells to propagate the infection. Reproduced with permission from Dow, J. [14].

Tetraviruses infect the midgut cells of Lepidopteran larvae in the wild and are notoriously difficult to replicate in cell culture with the only known example being PrV [11]. The general lifecycle of virus production in the wild is summarized in Figure 3. Release to the midgut exposes the virus to a highly alkaline environment (Figure 4) [14] that propagates the infection.

Tetraviruses are so-named due to their *T =* 4 icosahedral surface lattice. The *T =* 4 quasi-symmetry is seen in the nucleocapsids of enveloped viruses (e.g., Sindbis virus [16,17]), however tetraviruses are the only non-enveloped viruses reported with this T number. Tetravirus capsids have 4 copies of the capsid protein (Figure 1c) in the icosahedral asymmetric unit of the capsid and 240 copies of the same gene product in the complete virus capsid. These subunits are in four unique quaternary structure environments (Figure 1b). There is a significant repertoire of structural data available for tetraviruses, including high resolution X-ray models for two authentic viruses (NωV (PDB code 1OHF) [18], PrV (PDB code 2QQP) [19]), one virus-like-particle (VLP) (HaSV (PDB code 3S6P)), and cryoEM structures available for other family members. Comparing the structures of HaSV to NωV (70% sequence identity), demonstrates that the structure of VLPs is indistinguishable from authentic virus. SDS-PAGE analysis of the procapsid VLPs shows the presence of full-length protein subunits, while the capsid is composed of a large protein subunit (1–570) and a smaller peptide unit (571–644) that is the result of an auto-proteolysis event during maturation (Figure 1c).

NωV is the best-characterized member of the tetravirus family and is the focus of this review. We employ a baculovirus protein expression system [20] to generate particles that look and behave like the authentic virus, although they package primarily cellular RNA and messenger RNA for the capsid protein [21]. Hence, they are not infectious. Since the SF21 cells used for expression do not undergo the normal environmental changes of NωV infected cells (*i*.*e*., apoptosis), we purify procapsids from the expression system. X-ray and cryoEM structures of authentic virions were determined as well as cryoEM structures of VLP procapsid [22]. The availability of procapsids that can be matured *in vitro* led to a variety of biochemical and biophysical studies of maturation [15,23,24] and make NωV among the best model systems for the study of maturation.

## 3. Initial Assembly of NωV Procapsids

**Figure 5:**
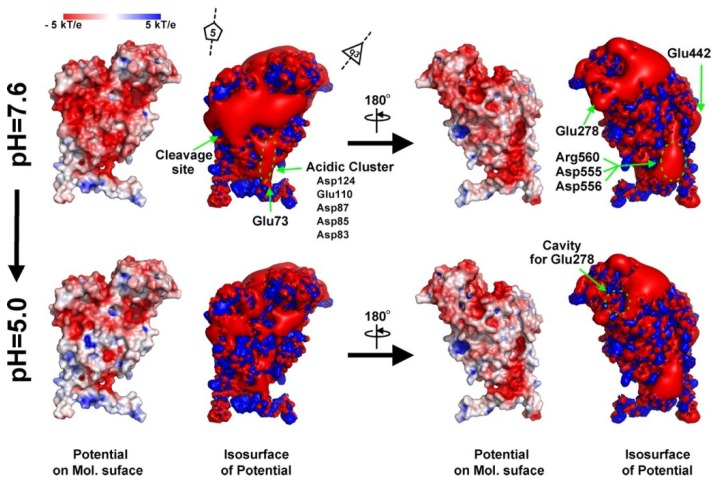
Electrostatic potential of an isolated A subunit at pH 7.6 and 5.0. The ±3-kT/e potential isocontours are shown as blue and red surfaces, respectively. The electrostatic potential was calculated for the monomeric protein, meaning that these potentials are not being influenced by the low-level dielectric environment or the charge distribution of neighboring subunits. Reproduced with permission from Matsui *et al*. [24].

NωV assembles with 240 copies of the 644-amino-acid capsid protein subunit into a porous, spherical procapsid that encapsidates the viral RNA genome. *In vivo*, this assembly takes place at neutral pH where the acidic surface of the subunits is negatively charged (Figure 5). These repulsive electrostatic forces maintain a 480Å diameter procapsid particle with the internal, disordered, positively charged domains (residues 1–45 and 625–644, containing 20 basic residues) interacting with RNA.

At the resolution currently available for the procapsid (8-Å cryoEM reconstruction) there is no discernable physical molecular switches present to guide proper *T =* 4 assembly. We hypothesize that the electrostatic surfaces act as a guide for proper subunit assembly. Such fields are an effective way of orienting subunits while avoiding the kinetic traps associated with strong protein-protein interactions. Subtle electrostatic anisotropy helps steer the subunits into the proper quaternary structure positions with minimal differentiation of quasi-equivalent subunits at this stage of assembly while reducing the possibility of arriving at false minima in the energy landscape. Thus procapsid assembly is in-tune with the pH of the local environment and the particle is poised for executing the maturation program.

## 4. Maturation: A Procapsid Becomes a Capsid

**Figure 6:**
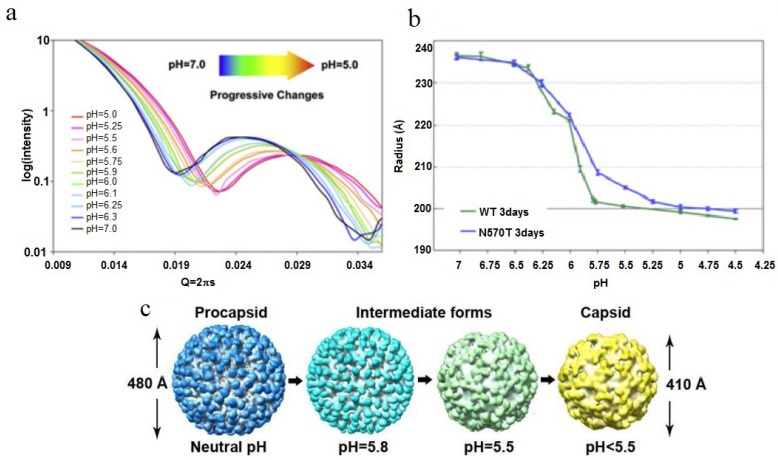
SAXS studies of the virus particle diameter as a function of pH. (**a**) SAXS measurement of the particle diameter as a function of pH shows a smooth continuum of particle sizes as the pH is adjusted; (**b**) At pH 5, both the NT and WT capsid have the same radius of approximately 205-Å; (**c**) CryoEM densities of several maturation intermediates, showing the change in particle diameter as a function of pH. Reproduced with permission from Matsui *et al*. [25].

The 480-Å diameter procapsid is roughly 20% larger than the mature 410-Å diameter capsid and is formed by full-length subunits. All subunits in fully mature particles have undergone auto-proteolysis at residue 571 creating the beta polypeptide (1–571) and gamma polypeptide (572–644). Gamma remains associated with the particle so there is no change in mass upon maturation. Cleavage is not initiated at neutral pH, however apoptosis, which occurs in the late stage of viral infection *in vivo*, reduces the pH of the cell to ~5, initiating maturation. Maturation can be performed *in vitro* by lowering the pH of the procapsid VLPs. This may be done in a stepwise fashion by setting specific pH values between 7 and 5 or by rapidly lowering the pH to 5. Protonation of the acidic protein surfaces reduces the negative charge and associated electrostatic repulsion allowing the subunits to approach each other, reducing the particle radius. The acidic residues at the interfaces neutralize at different pH values due to their different environments and SAXS studies have shown that a titration curve of the particle can be plotted with radius as the metric and that the overall pK_a_ for the particle is approximately 5.9 (Figure 6) [25]. The intermediate pH values act as “break points” in the execution of the maturation program code, allowing a detailed analysis of the subunit trajectories. Alternatively, stop-flow experiments revealed that the large-scale conformational change (LCC) takes place in less than 100 ms [26] if the pH is reduced to 5.0 instantaneously.

A change in pH alone does not yield a stable, mature capsid. If the pH is raised immediately following the pH drop, the virus particle will expand back to 480-Å although at a much slower rate (hours). If, however, ~10 min elapses before raising the pH, the particle will remain as the mature capsid at 410-Å diameter. Thus, a time-dependent event (auto-proteolysis at residue 570) acts as a locking mechanism to maintain the mature particle. The auto-proteolysis initiated by the LCC provides the locking mechanism that makes maturation irreversible. Remarkably, only 10% of the subunits must be cleaved to reach this stability indicating the highly cooperative nature of the symmetric particle. A proteolysis-defective mutant (N570T) will not lock the virus into a mature particle, but exhibits a reversible LCC, albeit with a large hysteresis [27]. A detailed analysis of auto-proteolysis following the first 10 min showed that the chemistry of the reaction proceeds with the same kinetics at pH 7 as it does at pH 5 implying that the pH dependent LCC creates the active site for the reaction that is otherwise pH insensitive between 5 and 7 [24].

**Figure 7:**
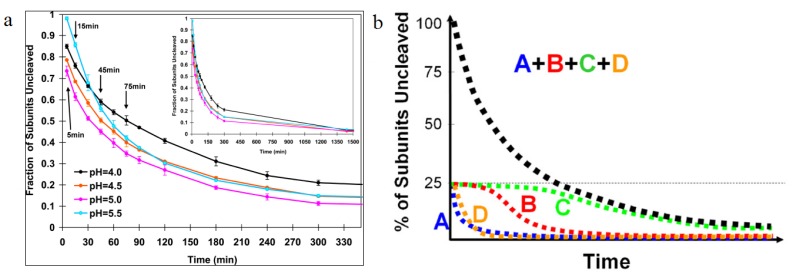
Autoproteolysis rates as a function of time. (**a**) The first appreciable amount of cleavage is observed 10 min after lowering the pH from 7.6 to 5. Half the subunits are cleaved at 30 min, then the cleavage slows dramatically; (**b**) A cartoon representation of cleavage as a function of time for individual subunits. Using difference maps from cryoEM experiments, the cleavage rate of individual subunits was determined. A and D subunits were shown to cleave quickly, with B and C subunits taking longer time to cleave, showing the dependence of subunit cleavage on the quaternary environment. Reproduced with permission from Matsui *et al*. [28].

**Figure 8:**
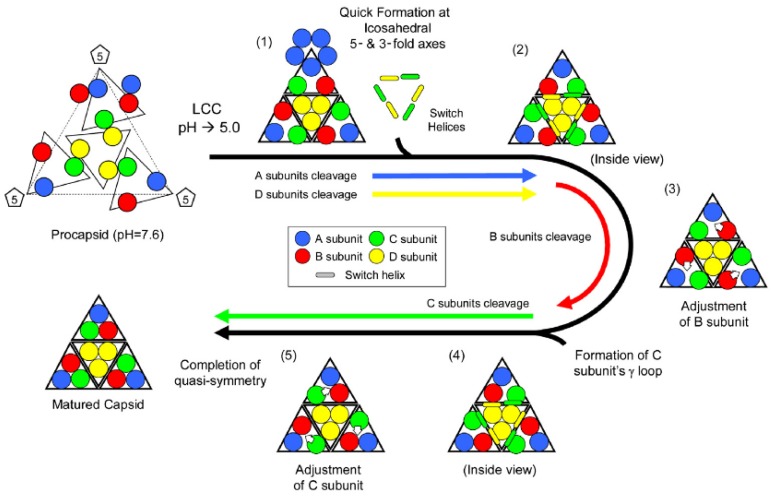
Proposed mechanism of subunit rearrangement during autoproteolysis. Initial LCC prompts quick cleavage in A/D. C/D switch helix interaction causes subtle changes that prompt B to cleave. B cleavage allows rearrangement in quaternary structure that promotes C to cleave, rearrange, yielding a mature faceted capsid with bent and flat contacts. Reproduced with permission from Matsui *et al*. [28].

All the active site residues and the cleavage site are on the same polypeptide indicating that the LCC must indirectly affect the catalytic site, subtly changing the positions of these residues by inter subunit contacts in their vicinity. Table 1 compares the residues in quaternary contact near the catalytic sites in the procapsid and capsid structures. The large difference in their positions relative to the active site demonstrates the potential role of the quaternary structure change on cleavage. Sufficient resolution of the procapsid is currently unavailable to directly detect the effect of these position differences, but they can be inferred by the observation that the subunits do not cleave at the same rate (Figure 7) probably because there are different quaternary interactions adjacent to the cleavage sites. The rapidly cleaving A-subunit site is dominated by 5-fold adjacent A-subunits and adjacent to the rapidly cleaving D subunit, there is an abundance of interactions from 3-fold related D-subunits. It is not surprising then that these subunits are the first to cleave because their quaternary environment is dominated by icosahedrally related interactions. In contrast the B and C subunits, which cleave much slower, primarily interact with quasi-related subunit types and molecular switches mediate the interactions related by quasi-symmetry axes. This leads to the current model summarized in Figure 8.

**Table 1:** Changes in the quaternary environment of NωV as a result of maturation. The ten closest amino acids from neighboring subunits are listed in order of distance (nearest distance listed first) for all subunits in both the procapsid and capsid.

Procapsid A	Capsid A	Procapsid B	Capsid B	Procapsid C	Capsid C	Procapsid D	Capsid D
I62-B	T444-A	L53-C	T444-C	I62-A	I62-D	L53-D	L57-D
V61-B	T63-B	A49-C	T63-A	V61-A	D60-D	S56-D	I62-C
T63-B	N443-A	V61-A	N443-C	T63-A	T63-A	A49-D	S56-D
F64-B	E442-A	S56-C	L57-C	T66-A	T444-D	L57-D	N60-C
T66-B	D448-A	V46-C	E442-C	F64-A	T63-D	E52-D	T63-C
D60-B	L445-A	A50-C	D448-C	P65-A	F64-A	A50-D	T444-B
A59-B	I62-B	E52-C	L445-C	N67-A	A59-D	G54-D	L53-D
D58-B	T449-A	N45-C	T449-C	A59-D	V61-D	A59-C	D58-D
P65-B	F64-B	L57-C	G566-C	S56-D	I62-A	V46-D	V61-C
V61-B	G566-A	T63-A	V61-A	N55-D	E103-B	N55-D	A59-C

**Figure 9:**
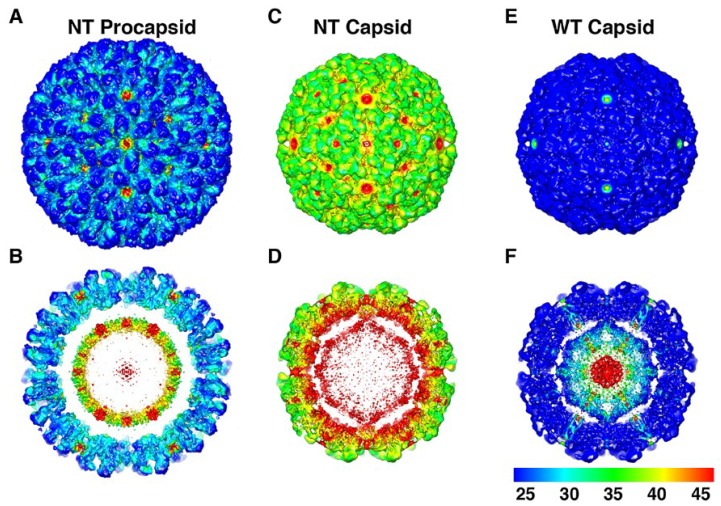
The structural fluctuations of the NωV particles plotted as standard deviation maps. Panels (**A**,**C**,**E**) are surface views of the cryoEM density maps of the NT procapsid, NT capsid, and WT mature capsid, viewed along an icosahedral 2-fold axis of symmetry and color coded according to values of their normalized standard deviation. The non-cleaving NT capsid and the mature cleaved WT capsid exhibit the highest and lowest standard deviations, respectively, and the NT procapsid is intermediate to these two. Panels (**B**,**D**,**F**) are the same as (**A**,**C**,**E**) but with the front halves of the density maps removed to reveal the standard deviation values for the internal features of the three particles. Reproduced with permission from Tang *et al*. [23]

A recent maximum likelihood analysis method for cryoEM reconstruction data allows a statistical description of the micro-states present in the ensemble of particles used for a reconstruction, providing a direct readout of the heterogeneity associated with any reconstruction in the form of variance maps [15]. Maturation intermediates were analyzed with this method [23] and the results clearly showed that the fully mature, cleaved, particle has the greatest homogeneity as evidenced by a dramatic reduction in the micro-states sampled when compared with the low-pH, compact form of the particle that had not undergone cleavage (NT). The pH 7.6 procapsid shows a moderate level of distinct microstates in the ensemble compared to the fully mature form, but far fewer than the NT capsid where there appears to be protein frustration as the particle samples a broad spectrum of microstates without reaching the ground state energy only reachable with the significant polypeptide rearrangement that occurs when cleavage occurs. (Figure 9).

Moderate resolution structural studies of the NT and mature capsid [22] show that residues 614–644 of the cleaved gamma peptides associated with the C and D subunits form a helical chock that functions to block the hinging of the C and D subunits at quasi 2-fold axes. Only with cleavage can these helices fully form and insert into the inter-subunit groove for the mature particle stabilization. This provides a clear structural role for cleaved gamma peptides associated with the C and D subunits. Gamma peptides from the A and B subunits, however, also undergo cleavage and clearly do not have a comparable role because of their different quaternary structure positions. Mature NωV particles exhibit lytic activity that is necessary for infectivity [29] that is also seen in the nodavirus Flock House virus (FHV) [30]. Recent studies have shown that the gamma peptide must be cleaved for this activity and that the pH must be at least 7.6 to activate the lysis of liposomes. The lytic activity is a maximum at 30 min post maturation when 50% of the subunits have undergone cleavage. Time-resolved cryoEM studies of maturation cleavage showed that the vast majority of subunits cleaved at 30 min are the A and D proteins. Because the gamma peptide of the D-subunit is involved in the chock, the lytic activity must be derived from the A-subunit gamma peptides. One gene product in NωV performs multiple activities in the mature particle depending on its quaternary structure position in the *T =* 4 lattice; D stabilizing the particle and A providing membrane -active lytic peptides with the same amino acid sequence. These studies demonstrate that this virus is a highly evolved machine that maximizes the function of a single gene product.

## 5. Conclusions

The NωV procapsid is a properly assembled particle (*i*.*e*., all subunits are correctly positioned on a *T =* 4 surface lattice) with minimal differences in local environments. Electrostatic forces at pH 7 guide procapsid assembly and minimize the possibility of kinetic traps, although the final assembly product is not robust. A drop in pH associated with cellular apoptosis initiates virus maturation—leading to a stable, infectious particle that is only released from cells very late in the initial infection. The LCC associated with the pH drop both initiates cleavage and forms differentiated local environments necessary for quasi-equivalent specific chemistries. The proteolysis product (gamma peptide) assumes two different roles depending on its associated subunit—either structural (C/D) or functional in the infection process (A). Events critical for the stability and function of the virus occur within 30 min of the pH reduction, although cleavage proceeds for more than 4 hours.

Maturation from procapsid to capsid appears to be a universal progression in animal viruses and dsDNA bacteriophage, but systems studied in each category behave differently suggesting that maturation is, in many cases, a convergent evolutionary process. HK97, a lambda-like dsDNA bacteriophage, shows a dramatically different maturation process when compared to NωV. HK97 subunits contain a 103-amino acid polypeptide at their N-termini that guides assembly (scaffolding domain) and it is digested by a packaged, virally encoded protease following assembly creating an empty capsid that is the substrate for the dsDNA packaging enzymes. This procapsid is metastable at pH 7 and undergoes a 2-state, stochastic, LCC when the pH is lowered to 5. The particle expands from 550 Å to 650 Å with a dramatic change in morphology and subunit contacts. Lowering the pH *in vitro* is thought to correspond to the dsDNA packaging *in vivo* that leads to maturation. Assembling a robust quasi-equivalent capsid requires at least one procapsid intermediate that serves an organizational role with weak, largely reversible interactions and an encoded program to confer stability and infectivity. This process can be performed in a variety of ways (convergent evolution) making virus maturation an intriguing area of biophysics and virus evolution. NωV is poised to provide fundamental new insights for non-enveloped animal viruses and given its progressive, readily controlled intermediates, may well provide a “movie” with the frames determined by pH.
